# Preparation, Characterization, and Performance of a Modified Polyacrylamide-Sericite Gel

**DOI:** 10.3390/ma16062524

**Published:** 2023-03-22

**Authors:** Haibo Jin, Xu Wang, Haizhong Yang, Guangxiang He, Xiaogang Li, Xiaoyan Guo, Lizhu Li

**Affiliations:** 1 State Key Laboratory of Shale Oil and Gas Enrichment Mechanisms and Effective Development, SINOPEC Research Institute of Petroleum Engineering, Beijing 102206, China; 2 Institute of Advanced Materials and Technology, University of Science and Technology Beijing, Beijing 102206, China; 3 Beijing Institute of Petrochemical Technology, College of New Materials and Chemical Engineering, Beijing 102617, China; 4 Beijing Key Laboratory of Fuels Cleaning and Advanced Catalytic Emission Reduction Technology, Beijing 102617, China; 5 Sinopec Shengli Oil Field Gudong Oil Production Plant, Dongying 257237, China

**Keywords:** PAM-sericite, plugging agent, blocking ratio, pressure resistance

## Abstract

In this study, a modified chemical plugging agent is prepared with the aim to reduce the well moisture content and improve the efficiency of oilfield development. In comparison to other chemical plugging agents, the composite gels plugging agents have excellent blocking capacity and erosion resistance. In this study, optimal conditions for the preparation of plugging agents were explored. The results showed that the performance of polyacrylamide-sericite (PAM-sericite) gel improved at a polymerization temperature of 60 °C, a crosslinker concentration of 0.5%, an initiator concentration of 0.75%, an acrylamide concentration of 10.0%, and a sericite concentration of 10.0%. The characterization of PAM-sericite gel showed a certain fold-like shape with a smoother surface, indicating that the doped sericite makes the plugging agent more compact and firm. It was also found that the blocking ratio of the plugging agent can potentially reach 99.5% after the addition of sericite. Moreover, failure stress of the skeleton structure and the water swelling degree were increased by 63.5% and 51.2%, respectively. Additionally, long-term stability, temperature resistance, pressure resistance and pressure stability also showed improvement to varying degrees. It was concluded that this gel has better stability against different kinds of salt solutions and is not affected by particle size.

## 1. Introduction

Most oil fields in China have gradually entered the medium-high water content period following a long-term industrial development. As a result of a further increase in the exploitation of the water injection process, the comprehensive moisture content of some oil fields has reached over 90%. Nevertheless, there is still a large amount of crude oil buried in the formation that remains unextracted. Therefore, it is pertinent to reduce the oil well water content to improve the efficiency of oilfield development. This rising interest is associated with oil increase in the oilfield development, coupled with the low cost, which has become an effective means to improve the effectiveness of the oilfield water injection development to enable the stable production of the oil reservoir [1,2,3]. To this end, there are two plugging methods, including mechanical and chemical methods. The mechanical water plugging method entails high cost and low efficiency. The chemical water plugging method is a more widely used method compared to the mechanical method due to its higher effectiveness. It entails a chemical reaction in the pore to produce chemical substances with a certain strength, which effectively blocks the permeable layer [4,5].

Scholars combined the polyacrylamide and other substance together to prepare a copolymer gel. Haraguchi et al. [6] used lithium alginate as inorganic particles, combined with a cross-linking agent, to prepare organic-inorganic composite hydrogel, which has high strength, uniform structure, good swelling performance and temperature resistance. Helvacloglu et al. [7] studied montmorillonite-polyacrylamide composite hydrogel, and the results showed that nano-montmorillonite sheets were evenly distributed in the modified hydrogel, showing good water absorption and swelling performance, and their mechanical strength and thermal stability were greatly improved. Compared with commercial polyacrylamide gels, this copolymer gel has better conductivity and salt resistance and temperature resistance, which can be applied to high reservoir heterogeneous oil fields.

Despite copolymer gel becoming the most commonly used system for water regulation and water plugging operations in recent years, it has certain shortcomings when applied to high-temperature and high-salt reservoirs, such as poor stability and low water plugging efficiency. In this regard, introducing high-temperature-resistant inorganic substances into the polymer gel system has become an effective method to improve the temperature and salt resistance of the water-conditioning agent. After introducing temperature-resistant inorganic substances into the polymer gel system, the performance of copolymer gels was significantly improved. Commonly used inorganic materials mainly include inorganic silicate [8], SiO_2_ [9,10,11], kaolin [12], laponite [13], bentonite [14], sepiolite [15], attapulgite clay [16], copolymers with comonomers having precursor moieties to form silica crosslinkers by a sol-gel process [17,18,19], etc. [20,21,22,23]. The above studies show that the addition of non-metallic mineral modifiers can help improve the salt and temperature resistance of composite materials to different degrees. He et al. [24] added inorganic silicate to the crosslinking reaction of polyacrylamide and phenolic resin. The author reported that the temperature resistance of the polymer gel system was greatly improved and could be stable at a high temperature of 130 °C for more than 60 days. Chen et al. [25] cross-linked the amide group of polyacrylamide through the silanol group of nanosilica through hydrogen bonding, which reduced the dehydration and contraction of the hydrogel while the stabilization time of the synthetic gel increased from 18 days to 180 days at 130 °C. Chen et al. [26] added nano-SiO_2_ to the polyacrylamide system; when the concentration of polyacrylamide was 0.18% and the concentration of SiO_2_ was 0.5%, the viscoelasticity and shear resistance of the SiO_2_-HPAM hybrid system were the largest, and the driving results showed that the recovery rate of SiO_2_-HPAM reached 28.7%, which was 6% higher than that of HPAM solution alone.

Therefore, this study selects high water old oilfield as the target reservoir, sericite as the inorganic substance, and acrylamide as the copolymer, to build a PAM-sericite copolymer plugging agent. The purpose of this study is to construct a gel-plugging agent with improved stability, salt resistance and temperature resistance, slow swelling effect, and high blocking rate. This will help solve the problems associated with plugging agents such as poor salt resistance and temperature resistance, low erosion resistance strength, and low blocking ratio.

## 2. Materials and Methods

### 2.1. Materials

Acrylamide (AM) and ammonium peroxydisulfate were provided by Beijing J&K Scientific Co., Ltd. (Beijing, China). Sodium bisulfite, sodium chloride, calcium chloride, magnesium chloride, and sodium dodecyl benzene sulfonate were provided by Sinopharm Chemical Reagent Co., Ltd. (Shanghai, China). N, N’-methylene diacrylamide (MBA) and sericite (3000 mesh) were provided by Shanghai Mindray Chemical Technology Co., Ltd. (Shanghai, China). The main chemicals and reagents used were of analytical grade.

### 2.2. PAM-Sericite Preparation

Add MBA and water to a three-mouth flask sequentially at room temperature, and stir until dissolved, then add sericite. The mixture was agitated with a water bath at a certain temperature. AM was added with N_2_ protection. After 4 h of reaction, PAM-sericite was obtained.

The total quality of the product was 30 g. The mass fraction of the crosslinker was referred to as the ratio of the MBA mass to the total mass of the product. The initiator mass fraction was the ratio of the mass of ammonium persulfate and sodium bisulfite used to the total mass of the product. The mass ratio of ammonium persulfate and sodium bisulfite was 1:1. The polymer monomer mass fraction was referred to as the ratio of the mass of AM used to the total mass of the product. The mass fraction of sericite was referred to as the ratio of the mass of sericite to the total mass of the product. The rest of the mass was water. The optimal preparation conditions for PAM-sericite were explored in the range of strips with the polymerization temperature at 40, 50, 60, 70, and 80 °C. Similarly, the range of crosslinker concentration was 0.25%, 0.50%, 0.75%, and 1.00%, while the initiator concentration was 0.50%, 0.75%, 1.00%, and 1.25%. The range for acrylamide concentration was, 7.5%, 10.0%, 12.5%, and 15.0%, while the sericite concentration was 7.5%, 10.0%, 12.5%, and 15.0%. The prepared gel PAM-sericite and polyacrylamide gel (PAM without sericite) were cut and broken by grinder. Particles were dispersed with industrial white oil and then dried in a vacuum drying tank. After that, particles were washed and dried with petroleum ether at room temperature, then were divided into three different particle sizes with sieves of <0.9 mm, 0.9–1.45 mm, and 1.45–2 mm. Particles with a particle size of 1.45–2 mm were used in the experiments. Three particles of different particle sizes were used in experiment in Section 3.3.3.

### 2.3. PAM-Sericite Characterization

#### 2.3.1. Scanning Electron Microscopy (SEM)

The gel PAM-sericite and PAM surface morphology were characterized by scanning electron microscopy (SEM) (SUPRA-55, ZEISS, Oberkochen, Germany) [27,28]. First, the plugging agent was placed on the sample seat, and the surface was sprayed with gold on the plugging agent after the vacuum. Photomicrographs were taken in very high vacuum conditions at 5 kV.

#### 2.3.2. Fourier Infrared Spectrometer (FTIR)

The molecular bond structure of the plugging agent was characterized by a Fourier infrared spectrometer (FTIR) (NICOLET6700, Seymour Fisher, Foxboro, MA, USA). The scan range was 400–4000 cm^−1^ with a resolution of 4 cm^−1^ for a total of 32 scans. Before the test, pure potassium bromide tablets and a deducted potassium bromide background peak were prepared to reduce interference.

#### 2.3.3. Universal Testing Machine (UTM)

The compression performance of the plugging agent was characterized by the universal testing machine (INSTRON 5982, Zwick Roell, Oberkochen, Germany). This analysis was carried out under a standardized method.

#### 2.3.4. Swelling Performance

The swelling performance of the plugging agent was mainly expressed by its water swelling degree [29]. The mass of the plugging agent before water absorption was recorded as *m*_0_, and the mass after water absorption was recorded as *m*, the water swelling degree was *C*, and the water swelling degree of the plugging agent was calculated by Equation (1):

(1) $$C(\%)=(m-m_{0})/m_{0}\times 100$$

#### 2.3.5. Blocking Ratio

The plugging experiment was performed in a salt solution with a total mineralization of 65,000 mg/L (NaCl 50,000, CaCl_2_ 10,000, MgCl_2_ 5000). The filling pipe was 25 cm long and 2.1 cm in diameter. Quartz sand was firstly used for filling, and then distilled water was introduced to reach a saturated state. The porosity was calculated from the mass difference before and after saturation of the sand filling pipe. A high-pressure pump was used for displacement, and after the pressure and flow rate were stabilized, the stable value was recorded and the permeability of the sand filling pipe was calculated. A certain mass of plugging agents was weighed and stirred slowly into a beaker of 500 mL saline solution at 60 °C. After swelling to a certain extent, it was poured into the device container, and the plugging agent granule solution was injected at a constant speed of 5 mL/min. The value was recorded after the pressure became stable. According to the permeability rate before and after the plugging, the blocking ratio was obtained, and the blocking ratio is shown in Equation (2):

(2) $$B(\%)=(k_{0}-k_{1})/k_{0}\times 100$$

where *B* is the blocking ratio, *k*_0_ is the permeability before the blocking, and *k*_1_ is the permeability after the blocking.

## 3. Results and Discussion

### 3.1. Preparation Conditions of the PAM-Sericite

Four variation levels of the five factors including polymerization reaction temperature, crosslinking agent concentration, initiator concentration, polymer monomer concentration, and sericite concentration were considered. The test mix ratio is shown in Table 1.

The experimental results were analyzed based on whether the polymerization reaction occurred and the water swelling degree. Firstly, the findings showed that when both the polymerization temperature and the concentration of the polymer monomer were too high or too low, the plugging agent could burst. Moreover, the polymerization time was too long and the brittleness and toughness of the polymer produced were too low. At this time, it was considered that no polymerization reaction occurred, and it was denoted by “−”, whereas a positive polymerization reaction would be indicated by a “+”. Secondly, a plugging agent prepared under suitable conditions was subjected to water absorption and swelling experiments in a salt solution with a total mineralization of 65,000 mg/L. It showed that the smaller the water swelling degree, the better the swelling properties of the plugging agent prepared under this condition.

In summary, under the preparation conditions of sample 10, the water swelling degree of PAM-sericite was minimal. Moreover, the PAM-sericite polymerization conditions were considered optimal when the polymerization temperature was 60 °C, the crosslinker concentration was 0.5%, the initiator concentration was 0.75%, the acrylamide concentration was 10.0%, and the sericite concentration was 10.0%. Under these preparation conditions, the PAM-sericite showed good swelling properties.

### 3.2. Characterization of the PAM-Sericite

The PAM-sericite prepared under sample 10 in Table 1 was characterized.

#### 3.2.1. SEM

**Figure 1:**
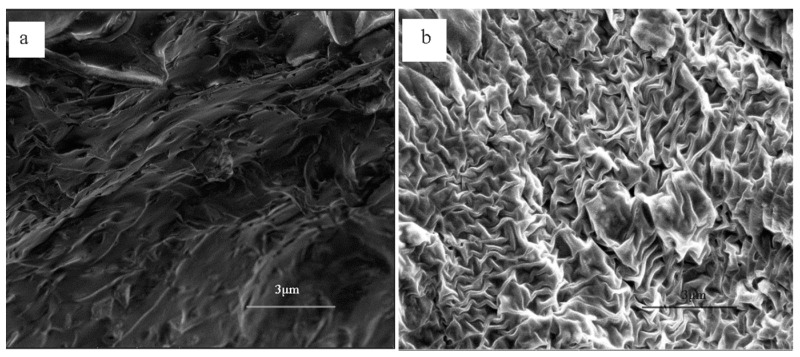
SEM image of plugging agent: (**a**) without sericite; (**b**) with sericite.

The prepared PAM-sericite was characterized using the SEM [30]. The surface of the PAM is irregularly flocculent and networked, indicating that acrylamide can form polymer chains to wind each other by polymerization (Figure 1a). Sericite itself has good toughness and elasticity, as well as high tensile and compression properties, which can be used as a good elastic filler. After the addition of sericite, it was seen that the surface of PAM-sericite was irregularly blocky and networked, with certain folds, and the surface was relatively smooth, indicating that the doped sericite made its skeleton more compact and firm (Figure 1b).

#### 3.2.2. FTIR

**Figure 2:**
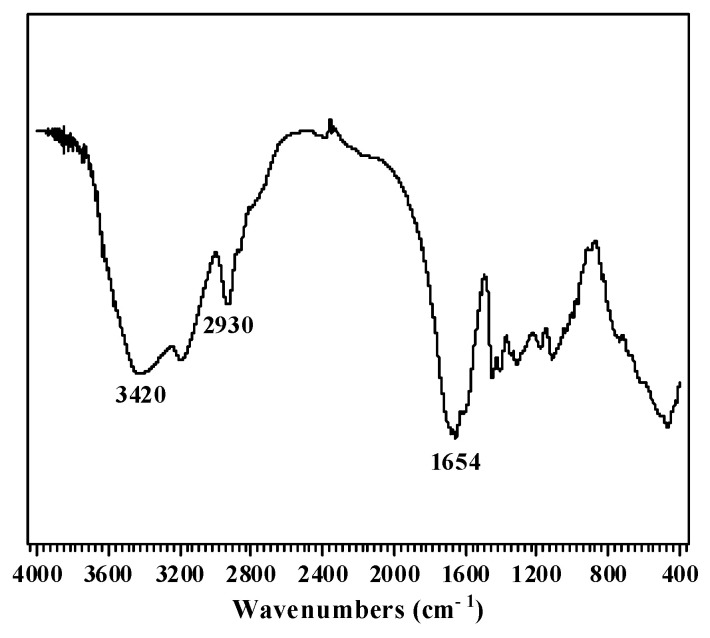
Plugging agent infrared spectra.

PAM-sericite was characterized using FTIR [31]. The characteristic peak at the 3420 cm^−1^ positions was the telescopic vibration of the amide group at N-H, the characteristic peak of 2930 cm^−1^ was the telescopic vibration of CH_2_ on C-H, and the characteristic peak at the 1654 cm^−1^ position was the C=O telescopic vibration (Figure 2). The characteristic peaks of the olefins did not appear on the map. The location of other characteristic peaks indicated that AM forms a polymer by crosslinking.

#### 3.2.3. Compressibility Test

**Figure 3:**
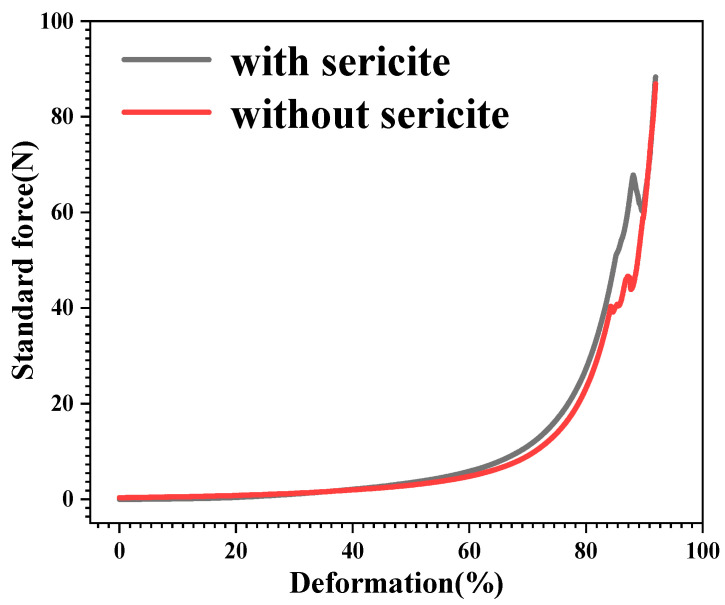
Compressibility test of plugging agent.

It was found that with increasing stress, the strain of the plugging agent continues to increase, mainly due to the deformation of its skeleton. When the stress reached 66.7 N, the skeleton structure of the plugging agent was destroyed, and the elastic deformation at this time became irretrievable. PAM (without sericite) suffered a stress of 40.8 N when the skeleton structure was destroyed (Figure 3). When the skeleton structure was deformed, the stress of PAM-sericite reached 63.5%, which was higher than the stress of the PAM plugging agent. This indicated that the toughness of the composite blocker after sericite doping was significantly improved.

### 3.3. Performance of the PAM-Sericite

#### 3.3.1. Swelling Properties

**Figure 4:**
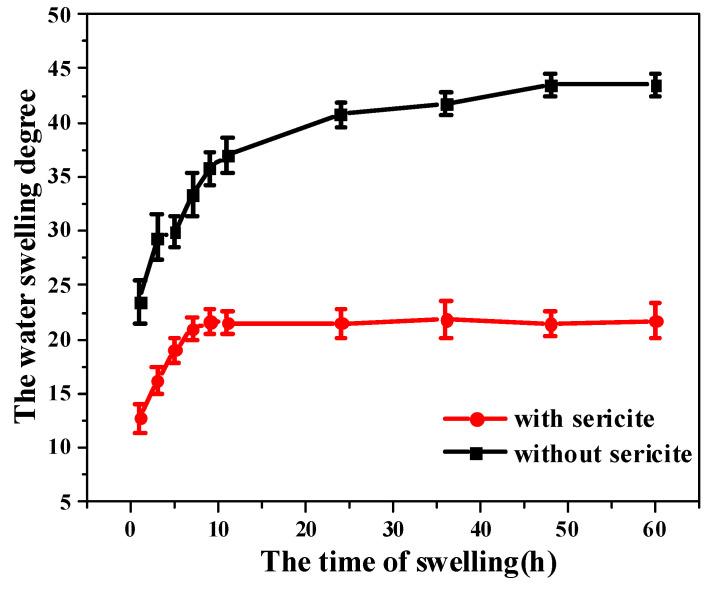
The water swelling degree of the plugging agent at room temperature.

The PAM-sericite and PAM plugging agents of the same quality were put into water for the water absorption expansion experiment at room temperature. The water swelling degree was calculated according to Equation (1). The results are shown in Figure 4.

It was found that the water swelling degree of the sericite-doped plugging agent was decreased by 51.2%, indicating that the doped sericite can effectively inhibit the water absorption swelling of the plugging agent. Therefore, it limited the water swelling degree of the plugging agent [32]. It was found that the addition of sericite improves the long-term stability and swelling properties of the plugging agent. In the early stage of swelling, water molecules mainly enter the composite plugging agent particles through physical adsorption and molecular diffusion. The water absorption and expansion were relatively fast. In the later stage of swelling, when the osmotic pressure inside and outside the composite plugging agent particles was zero, the swelling was balanced. Compared with PAM (without sericite), the water swelling degree of the composite plugging agent doped with sericite particles was significantly reduced, indicating that sericite particles could effectively inhibit the water absorption and swelling of composite plugging agent particles. The sericite particles adsorbed macromolecular chains and thus reduced their mobility, including the ability to unfold under the influence of water.

#### 3.3.2. Salt Resistance

**Figure 5:**
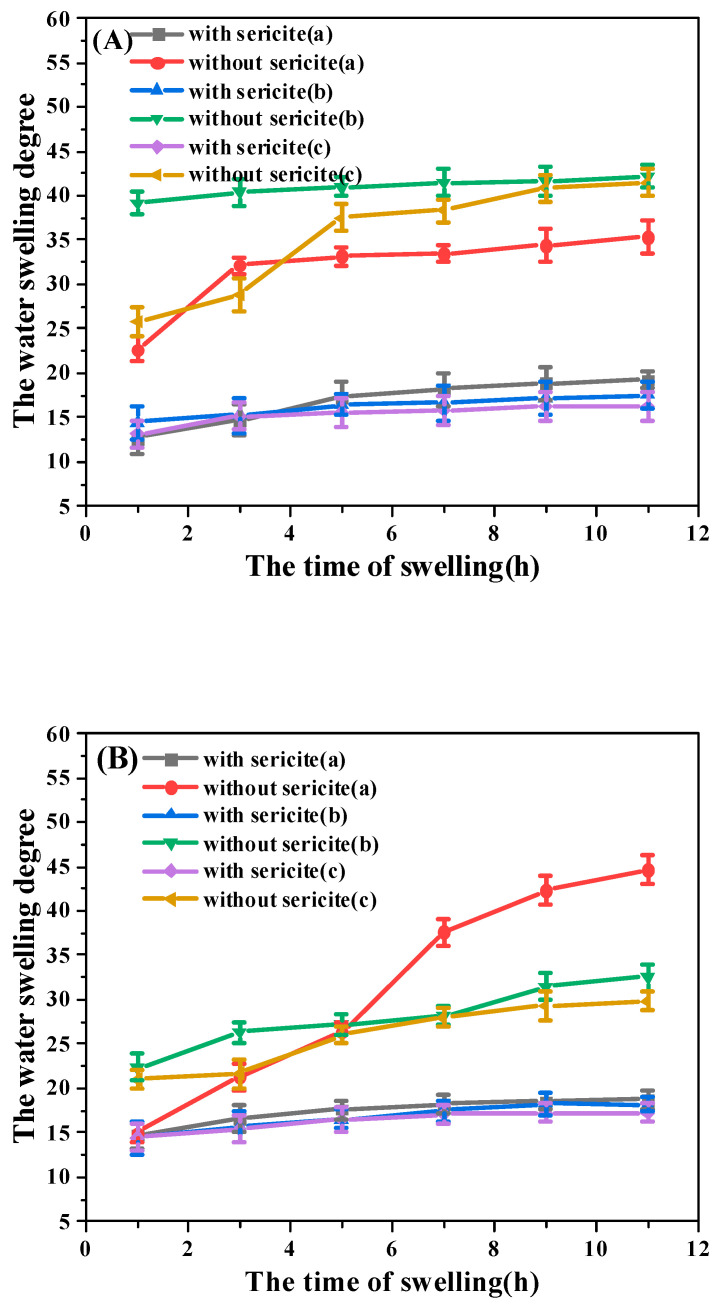

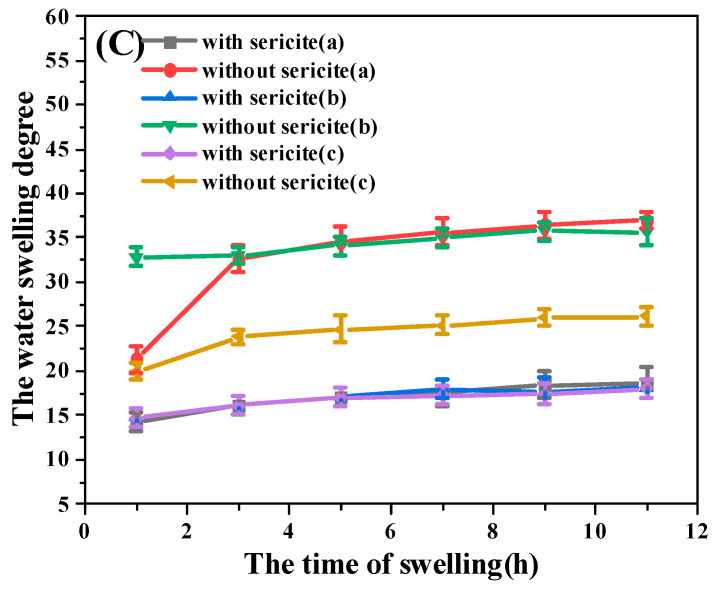
Swelling properties of the plugging agent in different solutions with different salt concentrations at room temperature. (**A**) NaCl solution: (a) 10,000 mg/L; (b) 30,000 mg/L; (c) 50,000 mg/L. (**B**) CaCl_2_ solution: (a) 1000 mg/L; (b) 5000 mg/L; (c) 10,000 mg/L. (**C**) MgCl_2_ solution: (a) 1000 mg/L; (b) 3000 mg/L; (c) 5000 mg/L.

The salt resistance of the plugging agent was tested by evaluating its swelling performance in the salt solution of different mineral abilities. It included NaCl with concentrations of 10,000, 30,000, and 50,000 mg/L, CaCl_2_ with concentrations of 1000, 5000, 10,000 mg/L, and MgCl_2_ with concentrations of 1000, 3000, and 5000 mg/L. The PAM-sericite and PAM plugging agents of the same quality were put into the salt solution for the water absorption expansion experiment at room temperature. The water swelling degree was calculated according to Equation (1). The results are shown in Figure 5.

It was found that the addition of sericite resulted in a reduced water swelling degree of the plugging agent under different salt solutions and different concentrations. After the addition of sericite, the water swelling degree of the plugging agent was stable between 10–20 times, indicating good stability and high salt resistance of the plugging agent under different kinds of salt solutions. The plugging agent was smaller than the water swelling degree of the CaCl_2_ solution under other conditions, which can be associated with higher Ca^2+^ ionic strength, resulting in the attenuation of the mutual repulsion between AM and sericite in the plugging agent [33].

#### 3.3.3. Particle Size Impact

**Figure 6:**
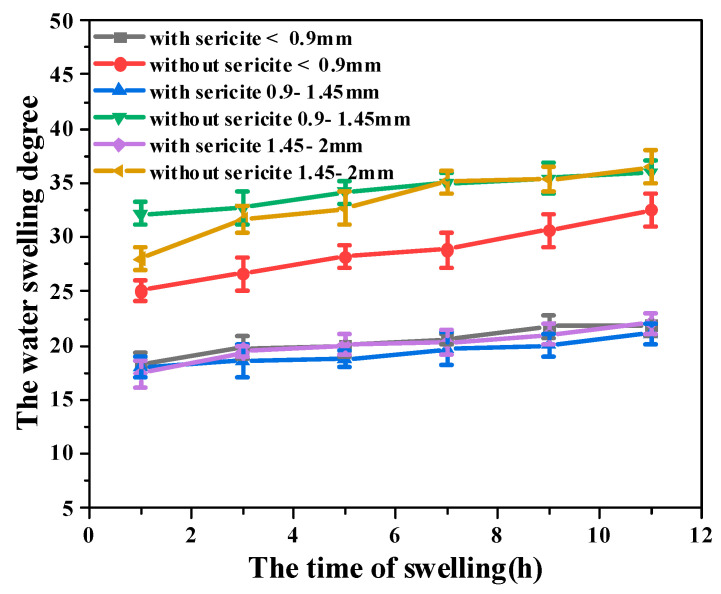
Swelling properties of the plugging agent in a different size at room temperature.

The particle size impact of the plugging agent was mainly reflected by its swelling performance at different sizes [34]. The PAM-sericite and PAM plugging agent were divided into three different particle sizes, including <0.9 mm, 0.9–1.45 mm, and 1.45–2 mm. The PAM-sericite and PAM plugging agents of the same quality were put into a mixed salt solution with mineralization of 65,000 mg/L for a water absorption expansion experiment at room temperature. The water swelling degree was calculated according to Equation (1). The results are shown in Figure 6.

It was found that after the addition of sericite, the water swelling degree of the plugging agent of different particle sizes is significantly reduced. Moreover, the water swelling degree of the plugging agent without sericite was greatly affected by the particle size, and the swelling degree was unstable; on the other hand, the swelling degree of the plugging agent with sericite was less affected by the particle size, and the swelling degree was stable. The results showed that the size of the plugging agent after adding sericite had no obvious effect on the water swelling degree. It can be explained by the fact that the particle size of the plugging agent without sericite is different, resulting in different specific surface areas and mass transfer areas of water through the interface, and therefore different expansion rates. Moreover, the expansion was not inhibited, which implies that the water swelling degree of particles of different sizes at a certain time point significantly varies [35]. In the case of the plugging agent with sericite, regardless of the particle size, it had the same water swelling degree (discussed in Section 3.3.1). Therefore, the relationship between the water swelling degree and particle size was sharply weakened.

#### 3.3.4. Temperature Resistance

**Figure 7:**
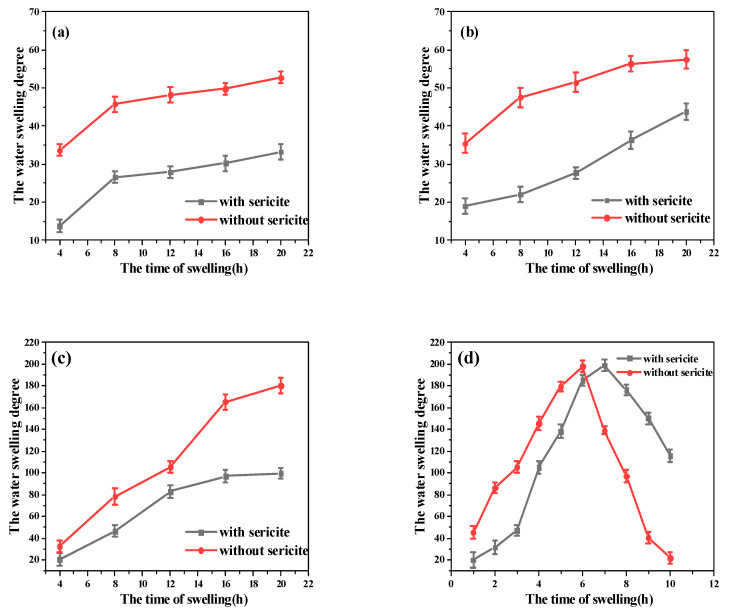
The swelling properties of the plugging agent in different temperatures: (**a**) 60 °C; (**b**) 90 °C; (**c**) 120 °C; (**d**) 150 °C.

The temperature resistance of the plugging agent was mainly reflected by its swelling performance at different temperatures [36]. To this end, the same quality of PAM-sericite and PAM plugging agents were subjected to temperature resistance experiments at 60 °C, 90 °C, 120 °C, and 150 °C. The water swelling degree was calculated according to Equation (1). The results are shown in Figure 7.

The addition of sericite enables a decrease in the water swelling degree of the plugging agent at different temperatures below 150 °C, indicating that the addition of sericite can improve the temperature resistance of the plugging agent. At 150 °C, the addition of sericite can increase the swelling time of the plugging agent.

#### 3.3.5. Blocking Performance

**Figure 8:**
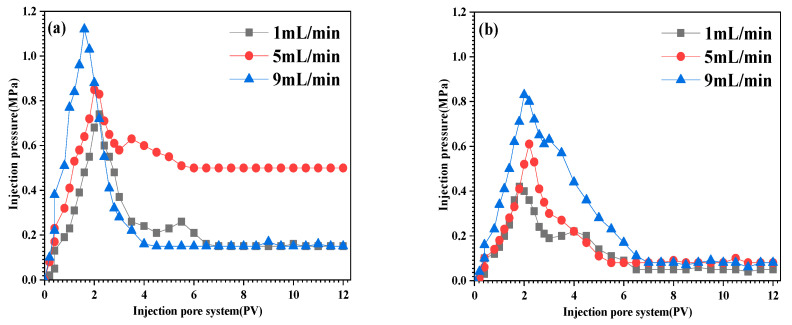
Blocking properties of plugging agent: (**a**) with sericite; (**b**) without sericite.

The blocking performance of the plugging agent was investigated using the sand filler replacement model. The results are shown in Table 2 and Figure 8.

It was found that under different flow rates, the blocking ratio of the plugging agent after adding sericite is effectively improved [37]. When the flow rate is 5 mL/min, the blocking ratio of the plugging agent is the largest. The blocking ratio of the plugging agent without sericite and with sericite is 97.4% and 99.5%, respectively, showing an increase of 2.1% (Table 2).

It was found that under different flow rates, the maximum plugging pressure and pressure stability of the plugging agent after adding sericite were significantly improved (Figure 8a). Moreover, when the flow rate was 9 mL/min, the plugging pressure of the plugging agent after adding sericite can reach up to 1.12 MPa. After erosion for a period of time, when the flow rate was 5 mL/min, the plugging pressure of the plugging agent after adding sericite can be stabilized at a maximum of 0.5 MPa. After a period of washing, the blocking pressure reached a stable value, indicating that after migration and shearing, the particles formed a stable bridging structure in the formation. Essentially, it showed that the particles no longer moved, and the pressure value tended to be stable. The stable pressure value reflects the stability and effectiveness of the sericite-based plugging agent. The maximum injection pressure and stabilization pressure of PAM-sericite were larger than those of PAM without sericite. This can be explained by considering the fact that the particles encounter sericite during shear crushing, preventing any further shearing. Furthermore, even after the PAM crosslink shear fails, the sericite particles can still achieve secondary plugging. It was concluded that the plugging agent after the addition of sericite has stronger pressure resistance and pressure stability.

## 4. Conclusions

In this paper, by doping sericite, the optimal preparation conditions of PAM-sericite composite plugging agent were determined, and the prepared composite plugging agent was granulated, and its swelling performance, salt resistance, and temperature resistance were explored. The optimal experimental conditions for the preparation of PAM-sericite is a polymerization reaction at a temperature of 60 °C, crosslinker concentration of 0.5%, initiator concentration of 0.75%, AM concentration of 10.0%, and sericite concentration of 10.0%. Through a performative investigation, it is shown that when the flow rate is 5 mL/min, the blocking ratio of the plugging agent is the largest, which can reach 99.5%. The failure stress of the skeleton structure and the water swelling degree are increased by 63.5% and 51.2%, respectively. It can be concluded that compared with the plugging agent without sericite, PAM-sericite have different degrees of long-term stability, temperature resistance, pressure resistance, and pressure stability. It has better stability against different kinds of salt solutions and is not affected by particle size.

## Figures and Tables

**Table 1:** Test mix ratio (water absorption expansion for 2 h in 65,000 mg/L mineralization solution).

Test Number	Polymerization Reaction Temperature (°C)	Crosslinker Concentration (%)	Initiator Concentration (%)	Polymeric Monomer Concentration (%)	Sericite Concentration (%)	Whether the Polymerization Reaction Has Occurred	The Water Swelling Degree
1	40	0.25	0.50	7.5	7.5	+	24.1
2	40	0.50	0.75	10.0	10.0	+	23.5
3	40	0.75	1.00	12.5	12.5	+	23.2
4	40	1.00	1.25	15.0	15.0	+	22.9
5	50	0.25	0.50	7.5	7.5	+	22.8
6	50	0.50	0.75	10.0	10.0	+	21.8
7	50	0.75	1.00	12.5	12.5	+	21.1
8	50	1.00	1.25	15.0	15.0	−	−
9	60	0.25	0.50	7.5	7.5	+	20.8
10	60	0.50	0.75	10.0	10.0	+	18.2
11	60	0.75	1.00	12.5	12.5	−	−
12	60	1.00	1.25	15.0	15.0	+	20.5
13	70	0.25	0.50	7.5	7.5	+	21.6
14	70	0.50	0.75	10.0	10.0	+	22.1
15	70	0.75	1.00	12.5	12.5	−	−
16	70	1.00	1.25	15.0	15.0	+	22.4
17	80	0.25	0.50	7.5	7.5	+	23.2
18	80	0.50	0.75	10.0	10.0	−	−
19	80	0.75	1.00	12.5	12.5	−	−
20	80	1.00	1.25	15.0	15.0	−	−
21	60	0.50	0.75	10.0	10.0	+	18.3

**Table 2:** Blocking performance of plugging agent.

Whether to Add Sericite or Not	Current Speed mL/min	Water Phase Permeability /10^−3^ μm^2^	Penetration Rate after Blocking/10^−3^ μm^2^	Blocking Ratio/%
with (1)	1	5716	129	97.7
with (2)	1	5876	141	97.6
without (1)	1	6762	207	96.9
without (2)	1	6678	214	96.8
with (1)	5	19,669	103	99.5
with (2)	5	19,775	119	99.4
without (1)	5	19,594	518	97.4
without (2)	5	19,628	510	97.4
with (1)	9	25,510	621	97.4
with (2)	9	25,540	690	97.3
without (1)	9	24,185	1164	95.2
without (2)	9	24,265	1189	95.1

## Data Availability

The data that support the findings of this study are available from the corresponding author upon reasonable request.
